# A Novel Approach for COVID-19 Patient Condition Tracking: From Instant Prediction to Regular Monitoring

**DOI:** 10.3389/fmed.2021.744652

**Published:** 2021-12-07

**Authors:** Evgeny A. Bakin, Oksana V. Stanevich, Mikhail P. Chmelevsky, Vasily A. Belash, Anastasia A. Belash, Galina A. Savateeva, Veronika A. Bokinova, Natalia A. Arsentieva, Ludmila F. Sayenko, Evgeny A. Korobenkov, Dmitry A. Lioznov, Areg A. Totolian, Yury S. Polushin, Alexander N. Kulikov

**Affiliations:** ^1^Raisa Gorbacheva Memorial Research Institute for Pediatric Oncology, Hematology and Transplantation, First Pavlov State Medical University, St. Petersburg, Russia; ^2^Research Department, Bioinformatics Institute, St. Petersburg, Russia; ^3^Department of Infectious Diseases and Epidemiology, First Pavlov State Medical University, St. Petersburg, Russia; ^4^Research Department, Smorodintsev Research Institute of Influenza, St. Petersburg, Russia; ^5^Department of Functional Diagnostics, First Pavlov State Medical University, St. Petersburg, Russia; ^6^World-Class Scientific Center, Saint Petersburg Electrotechnical University “LETI”, St. Petersburg, Russia; ^7^Center for COVID-19 Treatment, First Pavlov State Medical University, St. Petersburg, Russia; ^8^Department of Molecular Immunology, Saint Petersburg Pasteur Institute, St. Petersburg, Russia; ^9^Information Technology Department, First Pavlov State Medical University, St. Petersburg, Russia; ^10^Research Department, First Pavlov State Medical University, St. Petersburg, Russia; ^11^Clinic Management Department, First Pavlov State Medical University, St. Petersburg, Russia

**Keywords:** decision support systems, prognostic score, regular monitoring, COVID-19, SARS-CoV-2

## Abstract

**Purpose:** The aim of this research is to develop an accurate and interpretable aggregated score not only for hospitalization outcome prediction (death/discharge) but also for the daily assessment of the COVID-19 patient's condition.

**Patients and Methods:** In this single-center cohort study, real-world data collected within the first two waves of the COVID-19 pandemic was used (27.04.2020–03.08.2020 and 01.11.2020–19.01.2021, respectively). The first wave data (1,349 cases) was used as a training set for the score development, while the second wave data (1,453 cases) was used as a validation set. No overlapping cases were presented in the study. For all the available patients' features, we tested their association with an outcome. Significant features were taken for further analysis, and their partial sensitivity, specificity, and promptness were estimated. Sensitivity and specificity were further combined into a feature informativeness index. The developed score was derived as a weighted sum of nine features that showed the best trade-off between informativeness and promptness.

**Results:** Based on the training cohort (median age ± median absolute deviation 58 ± 13.3, females 55.7%), the following resulting score was derived: APTT (4 points), CRP (3 points), D-dimer (4 points), glucose (4 points), hemoglobin (3 points), lymphocytes (3 points), total protein (6 points), urea (5 points), and WBC (4 points). Internal and temporal validation based on the second wave cohort (age 60 ± 14.8, females 51.8%) showed that a sensitivity and a specificity over 90% may be achieved with an expected prediction range of more than 7 days. Moreover, we demonstrated high robustness of the score to the varying peculiarities of the pandemic.

**Conclusions:** An extensive application of the score during the pandemic showed its potential for optimization of patient management as well as improvement of medical staff attentiveness in a high workload stress. The transparent structure of the score, as well as tractable cutoff bounds, simplified its implementation into clinical practice. High cumulative informativeness of the nine score components suggests that these are the indicators that need to be monitored regularly during the follow-up of a patient with COVID-19.

## Introduction

As of May 2021, there were over 150 million confirmed cases of SARS-CoV-2 infection worldwide (1). Due to the frequent uncontrollable course of the infection, many medical workers are interested in creating an effective and flexible system for assessing the severity of patients' conditions. Such assessment systems are necessary both for an accurate prediction of treatment results and for the determination of indications for certain therapy regimens that may have multidirectional effects. Summarizing the experience of managing patients with COVID-19, the researchers came to the conclusion about the different power of various existing well-known scales for adverse outcomes prediction (SOFA, qSOFA, SAPS III, APACHE II) (2–4).

Also a few new scores focused on COVID-19 patients management appeared. Special attention was given to the tools using machine learning algorithms for assessing the risk of lethal outcome (5, 6). A large-scale analysis was previously performed by Clif et al. (7) using the QResearch database. This study examined several potential predictors of time to death and hospital admission due to COVID-19 and resulted in a few risk prediction algorithms including age, ethnicity, deprivation, body mass index, and comorbidities. In the studies considering blood biomarkers, several indicators demonstrated their potential for the prediction of outcome: aspartate aminotransferase (8), conjugated bilirubin (9), creatinine, urea (10), pro-calcitonin (11), C-reactive protein (12), and lymphocytes (13). These indicators are useful for death prediction both separately and in combination, as was shown via automatic feature selection tools based on logistic regression, support vector machine (SVM), random forest, etc. (14). However, despite all the advantages, the application of these scores in broad clinical practice remains very limited. We consider the following as main reasons for this:

Evaluation of some components of the scores may be complicated due to a high cost or the complexity of the measurement procedure. For example, the respiratory index included in well-known sequential organ failure assessment score (SOFA) can be calculated only by means of artery blood taken for an acid-base balance test (15).

1. Usually, in the published papers devoted to prognostic score development, the evaluation of a patient state is performed only once (e.g., at the moment of admission to the hospital or intensive care unit (ICU). However, the application of a score for regular patient monitoring is another important use case (e.g., for treatment strategy optimization), which requires an accurate examination of its temporal characteristics, such as the prediction range.
2. Since 2019, the pandemic landscape has been continuously evolving: new paradigms of treatment have replaced old paradigms, and more dangerous viral strains have appeared and spread widely. This makes it necessary to perform a regular validation of predicting approaches and their adjustment, if required.
3. Although the approaches based on state-of-the-art machine learning algorithms demonstrate a high accuracy (up to 90% and even higher), they frequently have a structure too complex for direct interpretation by a doctor. This complicates their acceptance in the medical community.

In this research, we aimed to develop and validate a novel prognostic score that has a transparent structure and is suitable for daily evaluation of a patient's condition. For this, its temporal characteristics, such as average prediction range, were carefully investigated. Due to existing discrepancies in definitions of various comorbidities, we decided to use only objective features, namely, anthropometry and blood tests. As additional requirements, we stated robustness, low cost, and wide availability of laboratory tests to be included in the score. This extends the scope of the index to non-ICU units as well as smaller regional clinics with a limited budget.

## Patients and Methods

### Study Design and Participants

**Figure 1:**
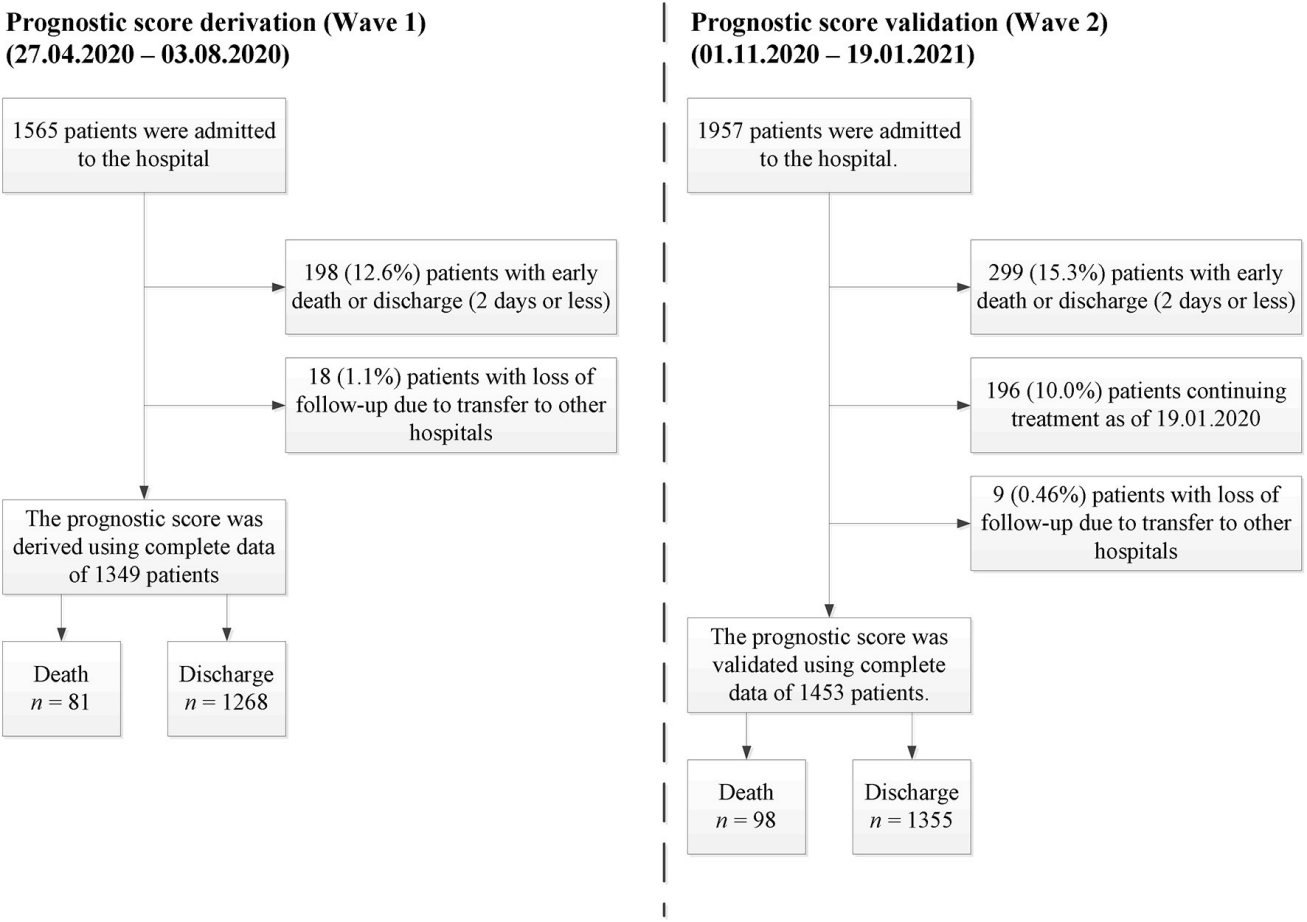
Study profile.

In this single-center cohort study, we used real-world data collected within the first two waves of the COVID-19 pandemic at St. Petersburg State Pavlov Medical University (Russia). Only patients over 18 years of age were treated in the hospital. The first wave data (collected 27.04.2020–03.08.2020) was used as a training set for the score development, while the second wave data (collected 01.11.2020–19.01.2021) was used as a validation set. No overlapping cases were presented in the study. For both cohorts, SARS-CoV-2 infection was proved using polymerase chain reaction (PCR). Before analysis, we excluded patients with a short follow-up period who died or were successfully discharged within the first 2 days of hospitalization. Thus, most patients with a moderate or a severe initial illness status were accepted for this research, while patients with a mild or a critical status were excluded (16). Patients who were lost to follow-up were excluded from this study as well. The overall study profile is given in Figure 1.

### Outcome of Interest

In-hospital death was chosen as the main event to predict. Since prediction must be timely, we evaluated not only sensitivity and specificity of the score, but also its prediction time range.

### Initial Features Set

In this research, we used the following initial feature set routinely gathered from hospital patients:

- *anthropometry*: age, sex, body mass index (BMI);
- *blood differential test*: total white blood cells (WBC), lymphocytes, neutrophils, monocytes, platelets, hemoglobin;
- *blood biochemical tests*: C-reactive protein (CRP), procalcitonin, creatinine, urea, total protein, sodium, potassium, lactate dehydrogenase (LDH), ferritin, conjugated bilirubin, aspartate aminotransferase (AST), alanine aminotransferase (ALT), troponin I, activated partial thromboplastin time (APTT), glucose, amylase, D-dimer, and fibrinogen.

Oxygen saturation (SpO2) was excluded from the list of potential predictors since it is difficult to measure saturation under equal conditions in patients with different stage of respiratory failure. For instance, SpO2 is measured in air in patients without oxygen support whereas in patients with severe respiratory failure such measurement may be risky as it requires disconnection from oxygen support.

All the data was downloaded from the local health information system (HIS) and manually checked for potential outliers. Off-scale values were set to the bound of an equipment dynamic range. There were no missing values in anthropometric data and only available blood tests data was used in the analysis. For the restoration of unknown values between two sequential tests, the “last observation carried forward” procedure (LOCF) was applied (17). To compare two cohorts, baseline values for the listed parameters were determined for all patients.

### Statistical Analysis

The analysis was performed based on all the available data from the described cohorts. The used data was manually checked for potential outliers (extremely large or low values of a feature). Typically, the detected outliers were caused by mixing up the units. Every presumable outlier was discussed with a data management department representative and fixed. Sex was coded with values of 0 and 1 for females and males, respectively. Continuous data was characterized by their medians and median absolute deviations (MAD) due to their non-normality (no data transformation was used). Categorical data was summarized as proportions. We compared baseline characteristics between the training and validation cohorts using the Mann-Whitney *U* test for continuous data and the χ^2^ test for categorical data. Cumulative incidence functions were compared with the Gray test. The correlations between the developed score and various cytokines were estimated with Spearman's rank coefficient. All *p*-values were two-sided, and all confidence intervals (CIs) were 95%. The study was conducted in adherence with TRIPOD statement (18).

To investigate the association between the dynamics of the features and outcomes, a 10-day period before an outcome (death or discharge) was analyzed. For each time-varying feature, we fit a robust linear regression model including the day before an outcome, an outcome itself, and their interaction as independent variables, whereas the feature value was a dependent variable (19). Thus, an interaction coefficient sign was used as an indicator of whether a feature increase or decrease was more typical in cases of expected lethal outcomes. Depending on the interaction coefficient sign, either an upper or a lower cutoff value was calculated for every feature. For static features (age, sex, and BMI), a day term and interaction term were excluded from the model, and a cutoff type was selected based on the outcome coefficient sign. When discussing the cutoff level, we did not find any unambiguous arguments for prioritizing either sensitivity or specificity, which is why this level was chosen to minimize their average.

Based on the conventional Bayesian approach (20), the resulting partial sensitivity and specificity of a feature were combined into a feature informativeness index (FII) as follows (see Supplement A for details):


$$\begin{array}{l} \text{FI}{\text{I}}_{\text{i}}=\text{log }\left[\left(\frac{\text{Sensitivit}{\text{y}}_{i}}{1-\text{Sensitivit}{\text{y}}_{i}}\right)\left(\frac{\text{Specificit}{\text{y}}_{i}}{1-\text{Specificit}{\text{y}}_{i}}\right)\right] \end{array}$$

Additionally, for every particular feature, we estimated a median prediction time range (defined as the time between the first true positive prediction for a patient and their death). Afterwards, a subset of features with the best informativeness and/or prediction range was selected to be combined into the proposed risk score (see section Results below). The score may be represented as a weighted sum of individual predictions made with a particular feature. The rounded values of FIIs were used as weights in the score. A detailed description of the rationales underlying the described procedure can also be found in Supplement A.

To validate the proposed score, we compared its sensitivity, specificity, and prediction range between the training and validation cohorts. To perform sensitivity analysis, the described pipeline was retrospectively applied in the reversed fashion: the score was trained using the second wave data and validated using the first wave data. A subgroup analysis was also performed, in which the score properties were analyzed for different age groups.

Additionally, the influence of the testing rate on the score was examined by means of the formation of a separate patient group (36 cases), in which every score component was intentionally tested not only according to a doctor's daily decision but also to ensure that the testing period was no more than 3 days. A quantile-quantile plot (QQ-plot) was used for the comparison of score value distributions in this group and in the control group, in which the testing rate was chosen in a conventional fashion (according to the doctors' daily decisions only). The control group was chosen with a propensity score matching algorithm based on baseline score component values. For the analysis of the relationship between a patient score and their individual mortality risk, we fit logistic regression with the following terms: maximal score during hospitalization, wave number, and their interaction. The second and third terms were included for the evaluation of the relationship robustness to the score application conditions changed between two waves.

Finally, we used the small amount of available data related to various cytokines tested occasionally since the beginning of the pandemic for the analysis of correlations between the score and cytokine levels. The correlations were estimated not only for the coinciding moments but also for a few time lags between the score calculation and cytokine testing. The aim of this part of our research was to evaluate the temporal interrelation between cytokine storm and subsequent development of multiple organ failure quantified with the developed score. It is worth noting that these cytokines were measured with a research purpose only and were not used for treatment strategy improvement (the measurements results were stored on the immunological laboratory server detached from HIS). Thus, the knowledge of their values could not influence further fluctuations of the proposed score caused by doctors' decisions.

## Results

### Comparison of Cohort

**Figure 2:**
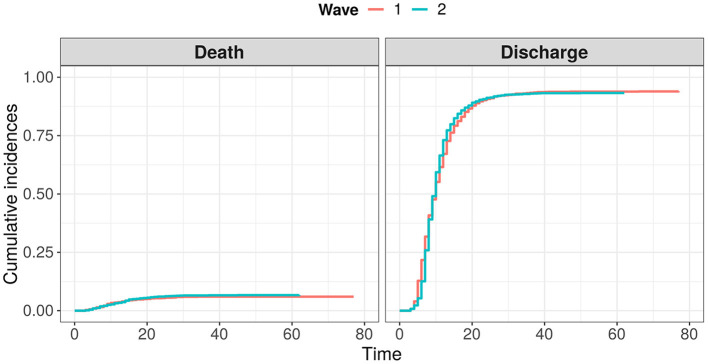
Comparison of cumulative distribution functions for events “death” and “discharge” between two waves of COVID-19.

The baseline cohort characteristics of the patients analyzed in the study are given in Table 1. A noticeable portion of patients from the considered cohorts have never experienced tests such as ferritin, pro-calcitonin, troponin I, and LDH. Moreover, a separate analysis showed that these tests were taken mostly for elderly patients and patients in a severe status. Hence, to avoid omitted variable bias, these features were excluded from further analysis. Comparing other features, we may conclude that even in cases with detected statistically significant differences, the clinical difference was considerably low. As a result, the differences between cumulative incidence functions for both events (death and discharge) were statistically insignificant (see Figure 2). However, we should note a slightly worse baseline condition of the 2nd wave patients on average.

**Table 1:** Baseline cohort characteristics.

**Test**	**Patients with known** **baseline values**			**Patients tested** **at least once**			**Average testing period** **(days)**			**Baseline values** median (MAD)^*^		
	**1**	**2**	***P***	**1**	**2**	***p***	**1**	**2**	***p***	**1**	**2**	***p***
Age	1,349 (100%)	1,453 (100%)	1	1,349 (100%)	1,453 (100%)	1	NA	NA	NA	58 (13.3)	60 (14.8)	<0.001
Sex	1,349 (100%)	1,453 (100%)	1	1,349 (100%)	1,453 (100%)	1	NA	NA	NA	F: 752 (55.7%) M: 597 (44.3%)	F: 752 (51.8%) M: 701 (48.2%)	0.029
BMI	1,349 (100%)	1,453 (100%)	1	1,349 (100%)	1,453 (100%)	1	NA	NA	NA	28.6 (5.34)	28.3 (5.12)	0.227
ALT	1,338 (99.2%)	1,446 (99.5%)	0.385	1,345 (99.7%)	1,452 (99.9%)	0.20	5.3	6.1	<0.001	30 (17)	30.2 (17.2)	0.496
Amylase	1,318 (97.7%)	1,440 (99.1%)	0.005	1,328 (98.4%)	1,447 (99.6%)	0.003	7.5	7.8	<0.001	57 (22.2)	57 (23.7)	0.547
APTT	1,316 (97.6%)	1,416 (97.5%)	0.961	1,326 (98.3%)	1,424 (98%)	0.579	6.9	7.6	<0.001	31.1 (4.74)	33 (5.19)	<0.001
AST	1,338 (99.2%)	1,446 (99.5%)	0.385	1,345 (99.7%)	1,452 (99.9%)	0.202	5.3	6.1	<0.001	36 (15.6)	37 (16.3)	0.220
Conj. bilirubin	1,325 (98.2%)	1,441 (99.2%)	0.038	1,334 (98.9%)	1447 (99.6%)	0.046	6.9	7.3	<0.001	2.5 (1.19)	2.5 (1.19)	0.969
Creatinine	1,339 (99.3%)	1,447 (99.6%)	0.367	1,346 (99.8%)	1,453 (100%)	0.111	5.4	5	0.014	0.088 (0.019)	0.089 (0.019)	0.159
CRP	1,339 (99.3%)	1,445 (99.4%)	0.693	1,348 (99.9%)	1,453 (100%)	0.481	2.4	2.2	<0.001	46 (54)	54.1 (54.6)	0.001
D-dimer	1,041 (77.2%)	1,263 (86.9%)	<0.001	1,241 (92%)	1,403 (96.6%)	<0.001	4.9	4.3	<0.001	577 (391)	519 (371)	0.008
Ferritin	799 (59.2%)	1,003 (69%)	<0.001	976 (72.3%)	1,269 (87.3%)	<0.001	7.2	6	<0.001	304 (259)	454 (372)	<0.001
Fibrinogen	1,304 (96.7%)	1,410 (97%)	0.644	1,319 (97.8%)	1,422 (97.9%)	0.897	6.6	8.8	<0.001	5.1 (1.59)	5.2 (1.5)	<0.001
Glucose	1,332 (98.7%)	1,445 (99.4%)	0.073	1,340 (99.3%)	1,451 (99.9%)	0.033	5.9	5.9	0.356	6.47 (0.98)	6.8 (1.3)	<0.001
Hemoglobin	1,348 (99.9%)	1,447 (99.6%)	0.157	1,349 (100%)	1,453 (100%)	1.000	2.6	2.5	0.001	139 (14.8)	137 (15.6)	0.010
LDG	740 (54.9%)	797 (54.9%)	1.000	962 (71.3%)	1,035 (71.2%)	0.967	6.4	6.3	0.146	268 (93.4)	265 (93.4)	0.965
Lymphocytes	1,348 (99.9%)	1,447 (99.6%)	0.157	1,349 (100%)	1,453 (100%)	1.000	2.6	2.5	0.001	1.2 (0.519)	1.1 (0.45)	<0.001
Monocytes	1,348 (99.9%)	1,447 (99.6%)	0.157	1,349 (100%)	1,453 (100%)	1.000	2.6	2.5	0.001	0.45 (0.193)	0.44 (0.2)	0.315
Neutrophils	1,346 (99.8%)	1,446 (99.5%)	0.405	1,348 (99.9%)	1,453 (100%)	0.481	2.6	2.5	0.001	3.77 (1.79)	4.21 (2.2)	<0.001
Platelets	1,348 (99.9%)	1,447 (99.6%)	0.157	1,349 (100%)	1,453 (100%)	1.000	2.6	2.5	0.001	203 (65.2)	214 (69.2)	<0.001
Potassium	1,340 (99.3%)	1,445 (99.4%)	0.878	1,346 (99.8%)	1,452 (99.9%)	0.357	5.4	5	0.008	4 (0.445)	4 (0.45)	0.019
Procalcitonin	892 (66.1%)	529 (36.4%)	<0.001	1,031 (76.4%)	665 (45.8%)	<0.001	5.2	7.2	<0.001	0.11 (0.058)	0.12 (0.06)	0.015
Sodium	1,340 (99.3%)	1,445 (99.4%)	0.878	1,346 (99.8%)	1,452 (99.9%)	0.357	5.5	5.3	0.149	139 (3.26)	138 (3.11)	<0.001
Troponin I	765 (56.7%)	356 (24.5%)	<0.001	910 (67.5%)	486 (33.4%)	<0.001	8.2	8.2	0.671	2 (2.97)	3 (2.97)	0.010
Total protein	1,333 (98.8%)	1,443 (99.3%)	0.240	1,342 (99.5%)	1,449 (99.7%)	0.372	7.3	7.5	0.012	72 (5.93)	71 (5.93)	<0.001
Urea	1,331 (98.7%)	1,443 (99.3%)	0.127	1,339 (99.3%)	1,449 (99.7%)	0.107	6.4	5.8	0.004	5.1 (1.93)	5.1 (1.93)	0.668
WBC	1,348 (99.9%)	1,447 (99.6%)	0.157	1,349 (100%)	1,453 (100%)	1.000	2.6	2.5	0.001	5.7 (2.09)	6.03 (2.28)	0.001

* *MAD, median absolute deviations; CRP, C-reactive protein; AST, aspartate aminotransferase; APTT, activated partial thromboplastin time; WBC, white blood cells; BMI, body mass index*.

### Evaluation of Features' Informativeness and Prediction Range

**Figure 3:**
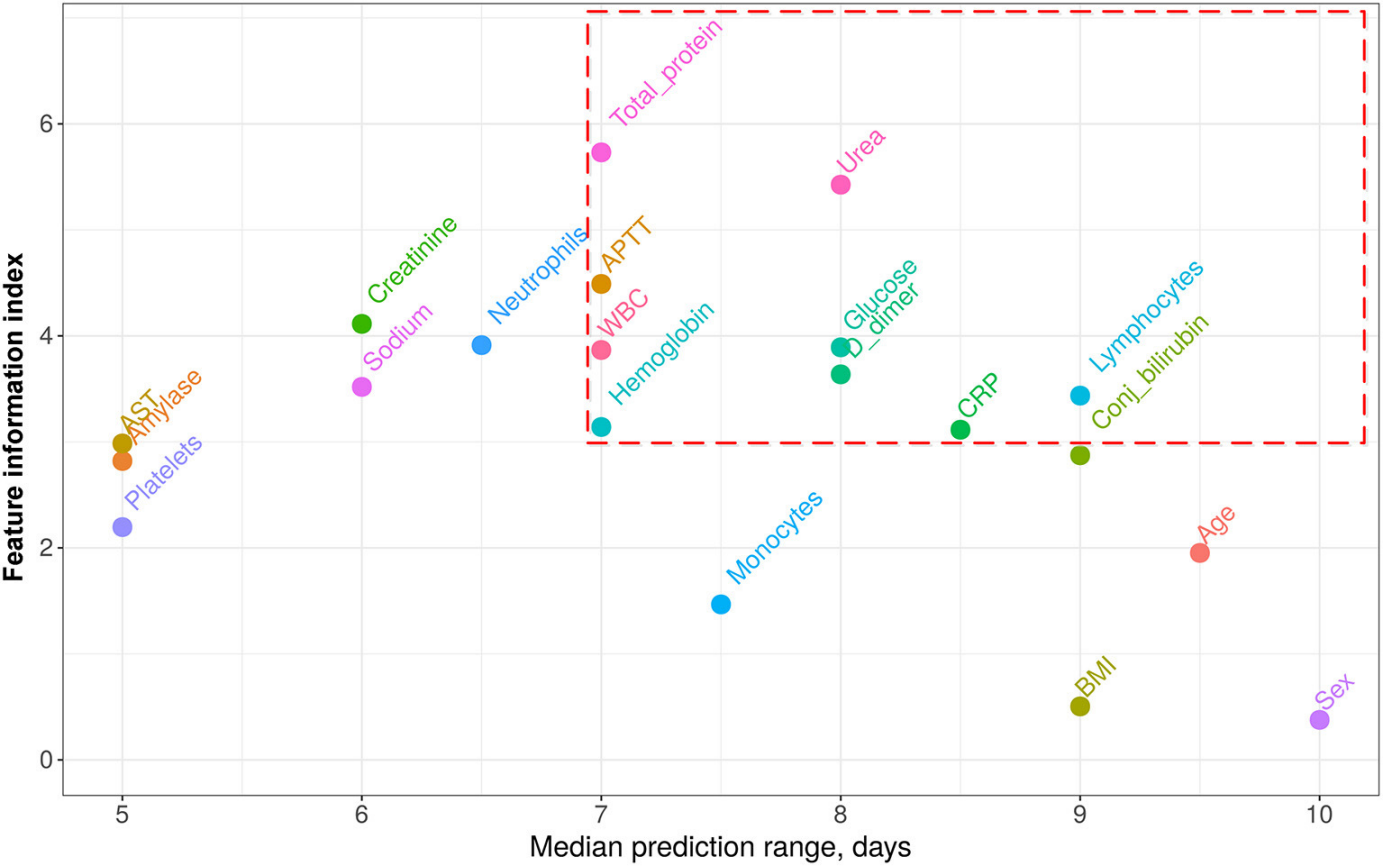
Features informativeness indexes (FII) vs. median prediction range. Features with prediction range ≥7 days range and weight ≥3 were accepted as the components of the proposed score (circled with red dashed line). CRP, C-reactive protein; AST, aspartate aminotransferase; APTT, activated partial thromboplastin time; WBC, white blood cells; BMI, body mass index.

Basing on robust regression results (see details in Supplement B), we accepted 20 features for the further analysis. Based on the first wave data, for each patient, we found the worst detected value of each of these features. These values allowed fitting a set of partial univariate prediction models (one per feature) and derive cutoffs, providing the minimum false positive rate (FPR), and false negative rate (FNR) arithmetic mean—(FPR+FNR)/2. Thus, for every feature, we calculated its partial prediction range, specificity, sensitivity, precision, and feature informativeness index (FII). An obtained range/informativeness trade-offs are depicted in Figure 3. The full summary for univariate analysis is given in Supplement B as well.

### Prognostic Score Statement and Its Validation

From all the features shown in Figure 3, we heuristically extracted only those with a median prediction range of at least 7 days and an information index of at least 3 (circled with the red dashed line). The 7 days range threshold was chosen since this period is usually sufficient to improve the treatment strategy and to try and reverse the course of the disease. Threshold 3 for FIIs was chosen to minimize the number of the score components and thus simplify its application. This resulting set of features with corresponding weights calculated as rounded FIIs is given in Table 2. The score value is obtained as a sum of weights of those features, which values crossed the specified threshold. The score values range from 0 to 36 points.

**Table 2:** The proposed score components.

**Feature**	**Threshold**	**Weight**
APTT	>42 s	4
CRP	>146 mg/L	3
D-dimer	>2,149 mg/L	4
Glucose	>9 mmol/L	4
Hemoglobin	<115 g/L	3
Lymphocytes	<0.7 × 10^9^/L	3
Total protein	<61 g/L	6
Urea	>11 mmol/L	5
WBC	>3.5 × 10^9^/L	4
Total score:		36 max

**Figure 4:**
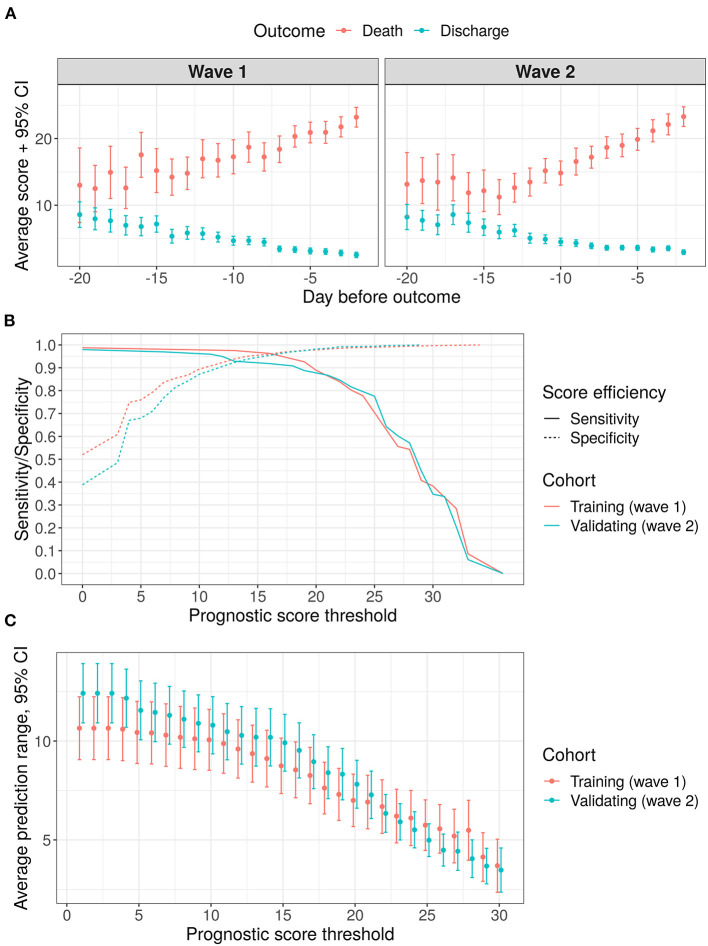
Results of score validation, points represent mean values, error bars−95% confidence intervals for the mean values **(A)** average score variation within 3 weeks before outcome: **(B)** sensitivity/specificity trade-off for various threshold levels; **(C)** prediction range dependency on a chosen threshold level.

Average score values per day among all the patients included in this research for both waves are shown in Figure 4A. The results of accuracy and promptness evaluation for the score are given in Figures 4B,C. As we see from Figure 4A the trends of averaged proposed score are almost linear both for improving and for worsening patients. It is worth noting that sensitivity and specificity are at least 90% for the validating sample with a threshold of 12 to 18 points (see Figure 4B). The average prognostic range for a lethal outcome ranges from 8.4 days (with a threshold of 18 points, 95% CI [7.1, 9.7]) to 10.3 days (with a threshold of 12 points, 95% CI [8.9, 11.7]). At the same time, sensitivity and specificity become equal at the level of 92% with a threshold of 13 points and an average prognostic range 10.2 days (95% CI [8.7, 11.7]).

**Figure 5:**
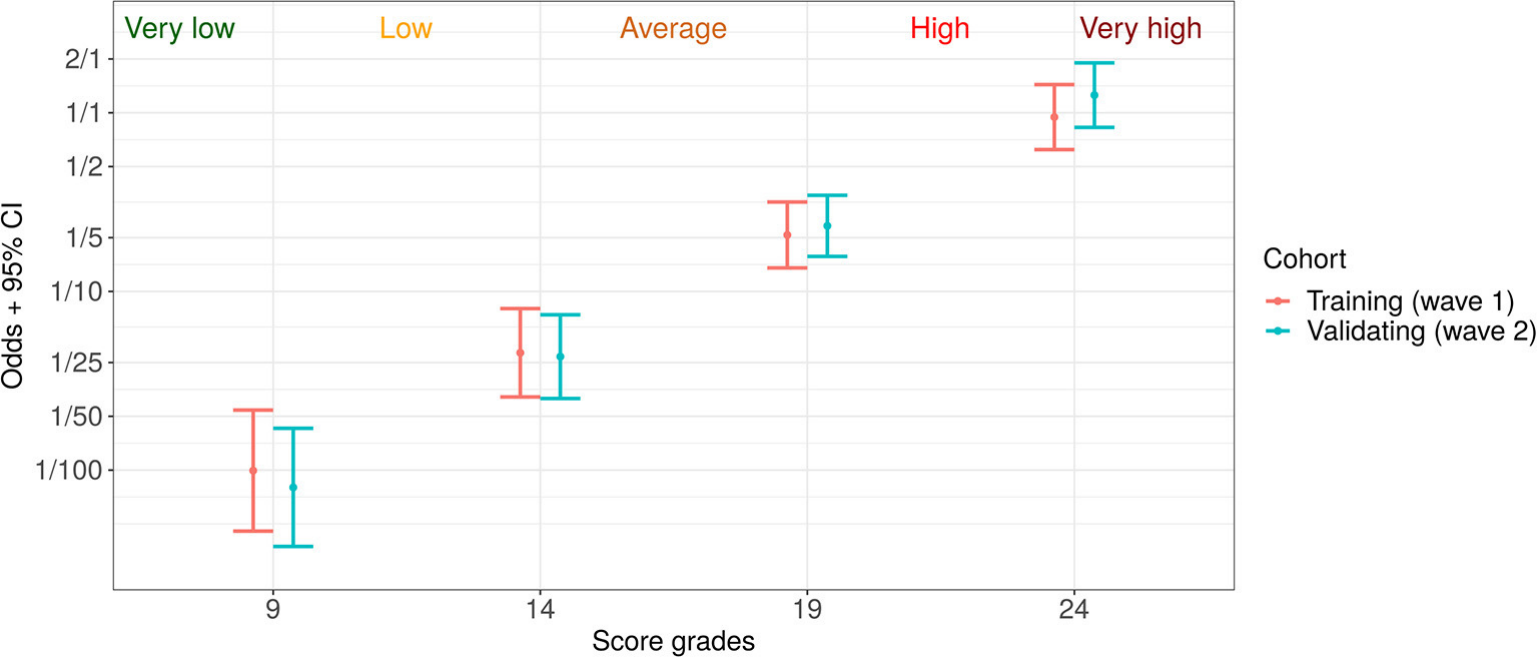
Five risk grades based on the proposed prognostic score: very low (death: discharge odds < 1:100), low (1:100–1:25), average (1:25–1:5), high (1:5–1:1), and very high (> 1:1).

For a simplification of patient individual risk estimation, we introduced the following five risk grades: very low, medium, high, and very high. Based on training cohort data, we adjusted the score bounds for their matching with expected death/discharge odds of 1:100, 1:25, 1:5, and 1:1. These bounds and corresponding odds with confidence intervals for both cohorts are given in Figure 5.

The results of a sensitivity analysis and a subgroup analysis are given in Supplements C, D. In brief, the score derived from Wave 2 data turned out to be similar to that described in Table 2. Actually, its components are a subset of the proposed score components (except WBC and total protein), and the overall characteristics are very similar to those reported in Figure 4 (both sensitivity and specificity are > 90% with expected prediction range > 7 days). A subgroup analysis was performed for two age groups (≥65 years old and <65 years old). As was shown, a senior age may potentially shift the statistical characteristics (namely, sensitivity and specificity) toward higher level of the score. One of the explanations of this finding is given in subsection analysis of the most noticeable prediction failures below. Also we noticed that in the senior age group the prediction range is typically smaller, which proves a faster course of the disease in this group and an additional requirement for attentiveness to senior patients.

### Score Application Examples

**Figure 6:**
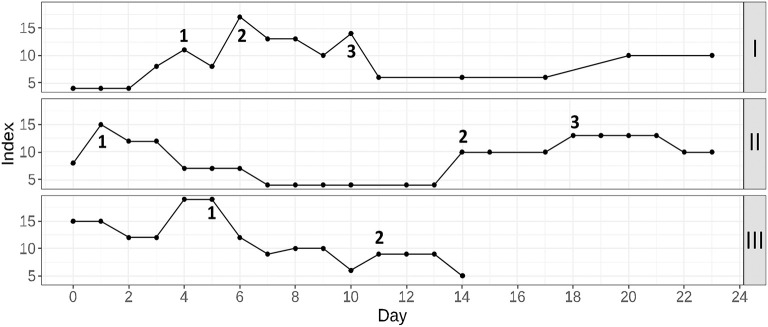
Three examples of the proposed prognostic score application in routine clinical practice. Patient I: 1, start of anticytokine + antibacterial therapy; 2, plasma exchange session; 3, non-invasive ventilation in the general observation unit; Patient II: 1, the moment of the beginning of glucocorticosteroid therapy; 2, discovery of the hematoma; 3, a discontinuation of anticoagulant therapy; Patient III: 1, start of anticytokine + antibacterial therapy; 2, start of additional antimycotic therapy.

In this subsection, three cases are given to illustrate a routine application of the proposed prognostic score. These cases were proposed by doctors as some of the most noticeable situations in which the score allowed to increase attentiveness in time. For all patients, COVID-19 pneumonia was diagnosed with a PCR test on hospital admission. The score variations for the considered cases are given in Figure 6.

Patient I (upper part of the figure) was admitted to the hospital with moderate respiratory failure (room-air SpO_2_−94%). Within the first 4 days of hospitalization, the patient's condition gradually worsened, as shown by increased lymphopenia, WBC, neutrophil, CRP, and ferritin levels. Moreover, glucose levels were poorly controlled. Therefore, therapy with tocilizumab was undertaken in combination with antibiotic therapy (point 1). This resulted in a general temporal improvement that included all the key laboratory parameters except for lymphocytes and ferritin. Room-air SpO_2_ remained at 89–90%, and the second CT scan was performed. A significant negative trend was found, and it was decided to transfer the patient to the intensive care unit (ICU) for a plasma exchange session and high-flow oxygen therapy (point 2). After this treatment, there were significant positive dynamics of clinical and laboratory parameters. The patient was transferred to the general observation unit, where the mitigation of respiratory failure was continued using non-invasive ventilation (point 3). Soon, the patient's condition stabilized, and he could be discharged home.

Patient II (middle part of the figure) was hospitalized with concomitant uncontrolled arterial hypertension and type 2 diabetes. She had severe respiratory failure (room-air SpO_2_-88–90%), extreme fatigue and unsteady gait. For correction of respiratory failure, glucocorticosteroid therapy (GCS) was undertaken (point 1), followed by infusion of immune anti-SARS-CoV-2 plasma and antibiotic therapy (due to the increased procalcitonin level). Then, clinical and laboratory improvement occurred. The target blood pressure was reached, and the oxygen saturation recovered to 94%. However, during preparation for discharge on the 13th day of hospitalization, sporadic growth of the score was detected. The discharge process was delayed and soon an unsteady gait as well as deterioration of respiratory function and increasing weakness. An examination of the patient showed a decrease in hemoglobin, accompanied by CRP elevation. During CT with contrast enhancement (point 2), the formation of a rounded area of fluid accumulation with smooth, clear contours in the left pectoralis minor was found. The lesion spread totally from the left axillary region to level IV-V of the sternocostal joint, with signs of unsharp infiltration of the surrounding tissue. The formation of a hematoma with an element of secondary inflammation was diagnosed. A discontinuation of anticoagulant therapy and maintenance therapy (point 3) were performed. When the patient's condition stabilized and improved, she was discharged.

Patient III (lower part of the figure) was admitted with respiratory failure that required glucocorticosteroid therapy from the first day of hospitalization. After a short-term condition improvement, a critical deterioration was detected by means of the proposed prognostic score. Because there was no progression of respiratory failure and the patient did not complain, it was difficult to assess his condition by means of daily physical examination. Given the high risk of poor outcome, anticytokine therapy with a JAK-kinase inhibitor in combination with antibiotic therapy was prescribed (point 1). Subsequently, a dramatic improvement in the patient's condition was observed. On the 11th day of hospitalization, additional antimycotic therapy was required due to colonization of the respiratory tract and oral cavity with Candida spp (point 2). Upon completion of the therapy course, the patient was discharged.

### Analysis of the Most Noticeable Prediction Failures

For the analysis of the score application limitations, we manually checked the most noticeable cases with prediction failure. In this dataset, three deaths occurred with a score value <9 points (“very low” risk grade). In all these cases, the deaths were caused by an extremely rapid deterioration: in two cases, they were associated with acute cardiovascular failure (for patients aged 60–65 and 80–85 years), and in the third case, they were associated with the development of subdural hematoma of the right hemisphere of the brain (for patients aged 90–95 years). Thus, there was not enough time to perform the laboratory tests and update the score.

Additionally, we considered 12 cases in which patients had score values <30 but survived and were discharged. In 5 of 12 patients, the observed decompensation was caused by prehospital therapy (in two cases, antibacterial therapy resulted in pseudomembranous colitis*;* in 3 cases, intensive anticoagulant therapy resulted in bleeding and the formation of hematomas). In 3 of 12 patients, the high score values reflected the progression of chronic diseases (stenosing cancer of the sigmoid colon, breast cancer, and polycystic kidney disease). At the same time, the course of COVID-19 was mild; thus, after effective management of chronic diseases, the patients were successfully discharged. Just in 4 of 12 patients, a severe course of COVID-19 was observed with significant impairment of respiratory function and the need to prescribe anticytokine therapy and non-invasive ventilation. This therapy successfully delayed the development of acute respiratory distress syndrome. Subsequently, the patients required the appointment of antibiotic therapy due to secondary bacterial complications after anticytokine therapy, which proved to be effective.

### Prognostic Score and Cytokines Landscape

**Figure 7:**
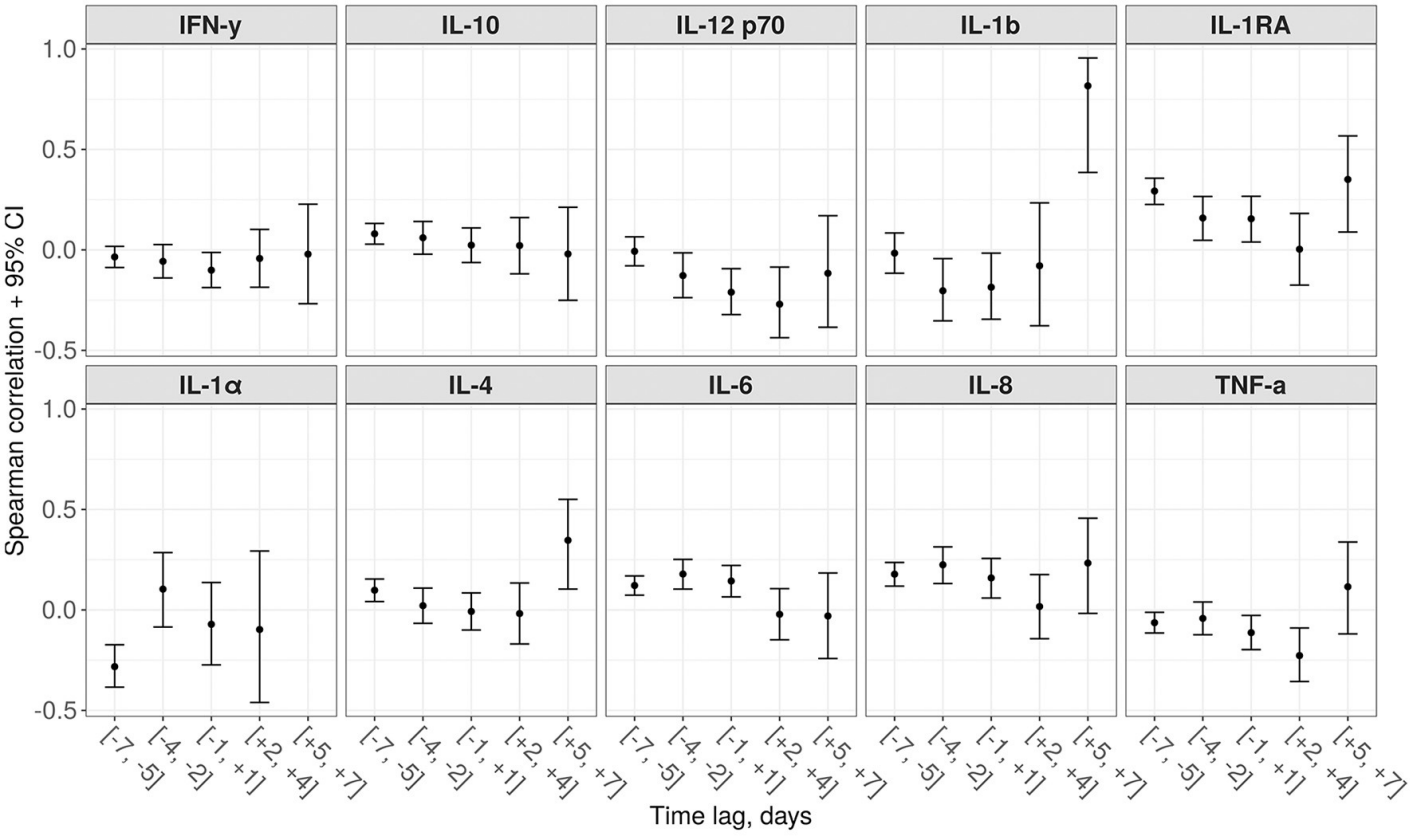
Spearman correlations between the proposed score and 10 various cytokines for different time lags (a negative lag represents cytokine testing preceding the score calculation, while a positive lag—on a contrary, succeeding).

In Figure 7, we present the results of the analysis of associations between various cytokine levels and the proposed score. The score value had a significant correlation with Interleukin-1 Receptor Antagonist IL-1RA (positive) and IL-1α (negative) measured 5–7 days before. For IL-6 and IL-8, the largest positive correlation was detected for the 3–5 days prior period.

## Discussion

The study resulted in development of a prognostic score that grades severity of condition in COVID-19 patients. Unlike other similar solutions, this score can be applied in real-time clinical practice, as proven by the study. We calculated the trade-off between sensitivity, specificity and prognostic range to realize this approach. As was shown in Results section, both validated sensitivity and specificity of 92% may be achieved with an average prognostic range of 10.2 days.

The score values range from 0 to 36 points. The selection of a certain threshold value in the score depends on the scope of its application. We assume that for every score range some reasonable guidelines may be proposed. Since COVID-19 is frequently accompanied by a fulminant course of the disease, every patient transition from one grade to a higher one (e.g., from the “very low risk” grade to the “low” grade) should lead to increased vigilance on the part of medical staff. For example, the deteriorations of cases described in subsection Score application examples were timely recognized thanks to the indicator of the current risk range introduced into the HIS. Further, for patients already admitted to hospital, the range from 14 to 19 shows an increasing negative risk and, as a result, indicates a necessity of an emergency specialist consultation. At the same time, the development of a similar score adapted for an outpatient management is also of great interest.

The comparison of the training and the validating cohorts demonstrates, that despite seasonal effects, change of paradigm in COVID-19 treatment and difference between baseline feature values, the proposed approach shows high accuracy and robustness. Thus, the differences in death: discharge odds between the considered cohorts for the selected risk grades were shown to be statistically insignificant (see Figure 4C). In addition, the analysis proved that the resulting values of the score are quite insensitive to the frequency of taking certain laboratory tests (see in Supplement Figure 4E). This effect can be attributed to the fact that an experienced attending physician is able to select an appropriate time interval between tests to keep the score up-to-date.

Another important benefit of the proposed score is simplicity of its internal structure. A comparison of 9 standard indicators with the thresholds and summarizing of the obtained weights is enough to calculate the score value. That is why the proposed approach, being potentially inferior in accuracy to more complex machine learning methods, is more preferable in routine clinical practice. Weights of indicators included into the score, reasonable threshold values and a clear reference to the time before the outcome allow attending physicians to support interpretation of the obtained values with their professional insights. The fact that most of the selected features were mentioned earlier as prognostic factors for COVID-19 patients further increases the interpretability of the score.

Thus, in research (21), urea together with creatinine reflects the state of kidney function at the time of COVID-19 illness, indicating a higher risk of severe infection in patients with chronic kidney disease (CKD) or acute kidney injury (AKI). In addition to indicating the current renal pathology and nephrotoxicity of the applied therapy, urea is one of the signs of catabolism in critically ill patients. The relationship between urea and catabolism has previously been demonstrated in patients with extensive trauma due to persistent muscle catabolism/rhabdomyolysis (22) and other severe conditions (23). The decrease in total protein can also be considered as an indirect reflection of both increasing renal dysfunction during COVID-19 in patients with CKD and “minor” hepatorenal syndrome (24, 25). High levels of CRP and ferritin are frequently considered criteria for the so-called cytokine storm (26). As the main feature of SARS-CoV-2 infection, this phenomenon also includes a concomitant increase in pro-calcitonin (27), which indicates a high concentration of pro-inflammatory cytokines with a simultaneous low level of interferon-gamma (28). In this situation, we have to deal with a differential diagnosis of secondary bacterial/fungal super-infection in COVID-19 patients. A low level of lymphocytes in the early stages of infection is considered a criterion for subsequent severity of the infection course (29). Cytokine storm together with lung injury are associated with a decrease in circulating lymphocytes both as a result of their direct depletion and their infiltration of the affected lung tissue (30).

A disturbance of glucose metabolism in patients with COVID-19 and its relationship with the severity of the course of infection may be the reflection of (i) poorly controlled diabetes in patients at the moment of hospitalization; (ii) a side effect of glucocorticosteroid therapy during infection; and (iii) more rarely—*de novo* development of diabetes as a consequence of SARS-CoV-2 infection (31). A decrease in hemoglobin in the early stages of the disease can be associated with a high concentration of circulating IL-6 and the risk of a cytokine storm (32). In the later stages of the disease, hemoglobin may be an indirect sign of a bleeding episode due to a massive anticoagulant therapy. Finally, coagulation abnormalities in COVID-19 patients are almost unavoidable satellites of this infection, leading to difficult-to-manage complications, such as venous thrombosis and thromboembolism (33). Thus, the mentioned features accepted as score components cover almost all the peculiarities of COVID-19 pathogenesis.

The statistically significant correlation between the score values and the level of some interleukins (in particular, IL-1α, IL-4, IL-6, IL-8, IL-1RA), taken 5–7 days before, demonstrates the expected time between the cytokine storm and development of multiple organ dysfunction syndrome. This suggests that in the future it will be possible to create accurate scores that include values of these interleukins as components and allow for even more long-term prognosis (34).

The performed sensitivity analysis allowed us to figure out the core score components, which presumably reflect the fundamental properties of COVID-19 pathogenesis, namely APTT, CRP, D-dimer, glucose, hemoglobin, lymphocytes and urea. These components together showed high informativeness and promptness for both waves. Despite the differences in seasons, treatment tactics and prevalences of various strains, this set of features provided stable sensitivity and specificity within both waves (see Supplement Figure C).

It is worth noting, that our research shows the potential for the accurate assessment of current patient state basing on routine blood tests only. Usually, similar approaches use such extra features as oxygen saturation, comorbidities, acid-base balance etc. However, their application may be complicated by the following practical aspects: impossibility of accurate SpO2 estimation in room air for the patients with respiratory failure, ambiguity in defining chronic diseases in various public health systems, injury risk of arterial blood sampling etc. Here we prove that a highly accurate score may be developed without them. This allows the scope of application to be extended to small regional hospitals, in which availability of various laboratory tests is limited. Finally, the described pipeline may be reproduced in other hospitals equipped with HIS. Thus, the score may be adapted to the local peculiarities.

## Study Limitations

The study has the following limitations. First, it was a single-center study based on a Russian population of persons over 18 years of age (mainly Caucasians) with a moderate to severe baseline illness status. Second, a few features that showed efficiency in previous studies (such as ferritin, LDH, procalcitonin, and troponin) were excluded from the analysis due to insufficient data. Third, the score did not contain any components reflecting a patient's respiratory function. Thus, an objective estimation of a patient's condition could be performed only in combination with oxygenation parameters. Finally, the experience of using the score in hospital showed that it can give misleading negative risks in case of noticeable progression of a chronic disease not related to COVID-19.

## Conclusion

The extensive testing of the score during the second wave of COVID-19 has demonstrated its attractiveness for application by medical staff due to precision in representing the current patient state and interpretability. From a practical point of view, the high cumulative informative value of the nine score components suggests that these are the indicators that need to be monitored regularly during the follow-up of a patient with COVID-19. Also, it was shown that the proposed score may be used as a tool for automatic detection of patients with dangerous deterioration and thus help reduce the burden on staff during high workload periods. Finally, due to the simplicity of the score composition methodology it can be easily adapted to any hospital with a standard health information system. As the most important extension of this research, we see an opportunity for external validation of the score in other hospital settings, ethnicities and age groups (35). Also, a careful accounting of concomitant chronic diseases progression by a doctor may further improve its interpretability and generalizability.

## Data Availability Statement

The datasets presented in this article are not readily available because of local ethic committee requirements. Requests to access the datasets should be directed to interdep@spb-gmu.ru.

## Ethics Statement

The study protocol was approved by the Biomedical Ethics Committee of I. P. Pavlov First Saint Petersburg State Medical University. The patients/participants provided their written informed consent to participate in this study. Written informed consent was obtained from the individual(s) for the publication of any potentially identifiable images or data included in this article.

## Author Contributions

EB performed statistical analysis and designed the proposed score. OS and EB implemented the software for the score visualization. LS and EK optimized the developed software and provided technical support. OS and MC conducted a literature review and performed medical writing. VBe and AB analyzed the score sensitivity to application conditions. GS and VBo analyzed practical limitations of the score in consultation with YP. NA and AT evaluated association between the score and a cytokines landscape. DL coordinated testing of the score. AK encouraged the team to develop the score and supervised the project. All authors discussed the results and commented on the manuscript.

## Funding

This work was supported by the Ministry of Science and Higher Education of the Russian Federation by the Agreement No 075-15-2020-933 dated 13.11.2020 on the provision of a grant in the form of subsidies from the federal budget for the implementation of state support for the establishment and development of the World-Class Scientific Center ≪Pavlov Center ≪Integrative physiology for medicine, high-tech healthcare, and stress-resilience technologies≫.

## Conflict of Interest

The authors declare that the research was conducted in the absence of any commercial or financial relationships that could be construed as a potential conflict of interest.

## Publisher's Note

All claims expressed in this article are solely those of the authors and do not necessarily represent those of their affiliated organizations, or those of the publisher, the editors and the reviewers. Any product that may be evaluated in this article, or claim that may be made by its manufacturer, is not guaranteed or endorsed by the publisher.
