# Supplementary material for: A Novel Approach for COVID-19 Patient Condition Tracking: From Instant Prediction to Regular Monitoring
Source: Front Med (Lausanne). 2021 Dec 7;8:744652. doi: 10.3389/fmed.2021.744652 (PMC8688846; doi:10.3389/fmed.2021.744652)
Supplement: Supplementary file 1 [file Data_Sheet_1.PDF]

Supplementary Material

A. A heuristic procedure for score components weights assignment

To optimize the score components weights, we assume that a set of *N* various laboratory tests is available. Each test *i* produces result X_i which may be either $\hat{D}_i$ in case of predicted death or $\hat{S}_i$ when successful discharge is predicted. Let α_i and β_i be test *i* marginal false positive and false negative error rates correspondingly. To draw a final decision basing on all the tests results, the following *a posteriori* (AP) probability ratio may be written:

$$AP\left(X_1\ldots X_N\right)=\frac{\Pr\left\{D\,|\,X_1\ldots X_N\right\}}{\Pr\left\{S\,|\,X_1\ldots X_N\right\}}=\frac{\Pr\left\{D\right\}\Pr\left\{X_1\ldots X_N\,|\,D\right\}}{\Pr\left\{S\right\}\Pr\left\{X_1\ldots X_N\,|\,S\right\}} \tag{A.1}$$

If a preliminary correlation analysis was performed and the given set of tests has a reasonably small mutual dependence, the following approximation of expression (A.1) may be proposed:

$$AP\left(X_1X_2\ldots X_N\right)\approx\frac{\Pr\left\{D\right\}\prod_{i=1}^{N}\Pr\left\{X_i\,|\,D\right\}}{\Pr\left\{S\right\}\prod_{i=1}^{S}\Pr\left\{X_i\,|\,D\right\}} \tag{A.2}$$

Let's split the products in the nominator and the denominator in (A.2) into two sub-products depending on marginal decisions results:

$$\frac{\Pr\left\{D\right\}\prod_{i=1}^{N}\Pr\left\{X_i\,|\,D\right\}}{\Pr\left\{S\right\}\prod_{i=1}^{S}\Pr\left\{X_i\,|\,D\right\}}=\frac{\Pr\left\{D\right\}\prod_{X_i=\hat{D}_i}\Pr\left\{X_i\,|\,D\right\}\prod_{X_i=\hat{S}i}\Pr\left\{X_i\,|\,D\right\}}{\Pr\left\{S\right\}\prod_{X_i=\hat{D}_i}\Pr\left\{X_i\,|\,S\right\}\prod_{X_i=\hat{S}_i}\Pr\left\{X_i\,|\,S\right\}}=$$

$$=\frac{\Pr\left\{D\right\}\prod_{X_i=\hat{D}_i}\Pr\left\{\hat{D}_i\,|\,D\right\}\prod_{X_i=\hat{S}_i}\Pr\left\{\hat{S}_i\,|\,D\right\}}{\Pr\left\{S\right\}\prod_{X_i=\hat{D}_i}\Pr\left\{\hat{D}_i\,|\,S\right\}\prod_{X_i=\hat{S}_i}\Pr\left\{\hat{S}_i\,|\,S\right\}} \tag{A.3}$$

The substitution of α_i and β_i instead of conditional probabilities in (A.3) results in the following expression:

$$\frac{\Pr\left\{D\right\}\prod_{X_i=\hat{D}_i}\Pr\left\{\hat{D}_i\,|\,D\right\}\prod_{X_i=\hat{S}_i}\Pr\left\{\hat{S}_i\,|\,D\right\}}{\Pr\left\{S\right\}\prod_{X_i=\hat{D}_i}\Pr\left\{\hat{D}_i\,|\,S\right\}\prod_{X_i=\hat{S}_i}\Pr\left\{\hat{S}_i\,|\,S\right\}}=\frac{\Pr\left\{D\right\}\prod_{X_i=\hat{D}_i}\left(1-\beta_i\right)\prod_{X_i=\hat{S}_i}\beta_i}{\Pr\left\{S\right\}\prod_{X_i=\hat{D}_i}\alpha_i\prod_{X_i=\hat{S}_i}\left(1-\alpha_i\right)}=$$

$$=\frac{\Pr\left\{D\right\}}{\Pr\left\{S\right\}}\prod_{X_i=\hat{D}_i}\left(\frac{1-\beta_i}{\alpha_i}\right)\prod_{X_i=\hat{S}_i}\left(\frac{\beta_i}{1-\alpha_i}\right)$$

The latter expression may be rewritten using indicator functions:

$$\frac{\Pr\{D\}}{\Pr\{S\}}\prod_{X_i=\hat{D}_i}\left(\frac{1-\beta_i}{\alpha_i}\right)\prod_{X_i=\hat{S}_i}\left(\frac{\beta_i}{1-\alpha_i}\right)=\frac{\Pr\{D\}}{\Pr\{S\}}\prod_i\left(\frac{1-\beta_i}{\alpha_i}\right)^{I\{X_i=\hat{D}_i\}}\left(\frac{\beta_i}{1-\alpha_i}\right)^{I\{X_i=\hat{S}_i\}}=$$

$$=\frac{\Pr\{D\}}{\Pr\{S\}}\prod_i\left(\frac{1-\beta_i}{\alpha_i}\right)^{I\{X_i=\hat{D}_i\}}\left(\frac{\beta_i}{1-\alpha_i}\right)^{1-I\{X_i=\hat{D}_i\}}=\frac{\Pr\{D\}}{\Pr\{S\}}\prod_i\left(\frac{\beta_i}{1-\alpha_i}\right)\left[\left(\frac{1-\beta_i}{\alpha_i}\right)\left(\frac{1-\alpha_i}{\beta_i}\right)\right]^{I\{X_i=\hat{D}_i\}}$$

Applying a logarithmic function to the AP ratio gives:

$$\log AP(X_1\ldots X_N)=\log\frac{\Pr\{D\}}{\Pr\{S\}}+\sum_i\log\left(\frac{\beta_i}{1-\alpha_i}\right)+\sum_i I\{X_i=\hat{D}_i\}\log\left[\left(\frac{1-\beta_i}{\alpha_i}\right)\left(\frac{1-\alpha_i}{\beta_i}\right)\right] \tag{A.4}$$

A conventional classification approach consists in comparing $\log AP(X_1\ldots X_N)$ with a threshold chosen according to a required sensitivity/specificity trade-off. As we can see, the first two components of the sum in (A.4) don't depend on values $X_1\ldots X_N$. Thus, they may be omitted without loss of prediction efficiency. It is also worth noting, that the expression given in rectangular brackets in (A.4) coincides with an odds ratio. The resulting score function will be written then as follows:

$$Score=\sum_i I\{X_i=\hat{D}_i\}\omega_i\,.$$

Here:

$$\omega_i=\log\left[\left(\frac{1-\beta_i}{\alpha_i}\right)\left(\frac{1-\alpha_i}{\beta_i}\right)\right]$$

In the above equation [.] denotes a rounding procedure.

B. Univariate analysis

Table B.1 Robust regression coefficients for considered features

Feature	Intercept	Day before outcome	Lethal outcome	Interaction	Interaction *p*-value	Threshold_type
Age	55,561	-	17,24004	-	-	Upper
ALT	62,013	1,792829	-6,73908	-0,45107	0,277375	Lower
Amylase	65,096	0,401711	7,440098	1,897953	2,8E-06	Upper
APTT	30,931	-0,15563	23,48463	1,847344	7,92E-63	Upper
AST	43,385	-0,04688	28,82057	2,487885	1,68E-14	Upper
BMI	29,069	-	-0,85677	-	-	Lower
Conj_bilirubin	2,272	-0,04336	1,795281	0,123558	7,79E-12	Upper

Creatinine	0,081	-0,00053	0,074193	0,00656	3E-83	Upper
CRP	16,446	-3,84408	113,1996	5,847141	3,39E-29	Upper
D_dimer	875,320	-9,03413	3386,114	210,0928	1,67E-51	Upper
Ferritin	420,134	-1,10278	422,527	1,478446	0,886502	Upper
Fibrinogen	4,840	-0,04959	0,083639	-0,01128	0,656259	Lower
Glucose	6,361	-0,05777	3,12614	0,055615	0,043945	Upper
Hemoglobin	131,372	-0,23233	-28,2304	-0,59104	0,003274	Lower
LDH	246,656	-8,00863	428,0746	27,90315	5,88E-41	Upper
Lymphocytes	1,737	0,050565	-0,98658	-0,05384	1,45E-18	Lower
Monocytes	0,608	0,011606	-0,11799	-0,01207	1,64E-05	Lower
Neutrophils	3,822	-0,10006	8,764783	0,46006	2,86E-47	Upper
Platelets	313,745	7,587005	-143,614	-13,7101	2,41E-35	Lower
Potassium	4,477	0,036149	0,232668	0,007987	0,259182	Upper
Procalcitonin	0,098	-0,00445	1,39404	0,112736	0	Upper
Sex	0,451	-	0,086531	-	0,521291	Upper
Sodium	141,021	0,185574	4,88449	0,244052	6,48E-07	Upper
Toponin I	0,002	-0,00021	0,056907	0,004587	7,1E-179	Upper
Total_protein	66,428	-0,21607	-13,8551	-0,34804	0,001211	Lower
Urea	5,291	-0,07739	16,97786	1,107044	2,9E-112	Upper
WBC	6,462	-0,0296	7,680757	0,387139	5,59E-28	Upper

Table B.2 Partial prediction effectiveness of the features

Feature	Sensitivity	Specificity	Precision	Threshold	Threshold type	FII	Median prediction range, days
Age	0.59	0.83	0.18	68	Upper	1.95	9.5
Amylase	0.68	0.89	0.28	104	Upper	2.82	5
APTT	0.93	0.88	0.32	42.4	Upper	4.49	7
AST	0.80	0.83	0.23	83	Upper	2.98	5
BMI	0.41	0.71	0.08	25.95	Lower	0.50	9
Conj. bilirubin	0.88	0.71	0.16	3.3	Upper	2.87	9
Creatinine	0.85	0.91	0.39	0.123	Upper	4.11	6
CRP	0.84	0.81	0.22	146	Upper	3.11	8.5
D-dimer	0.86	0.86	0.28	2149	Upper	3.64	8
Glucose	0.91	0.82	0.25	8.9	Upper	3.89	8
Hemoglobin	0.84	0.82	0.23	114.5	Lower	3.14	7
Lymphocytes	0.85	0.84	0.26	0.7	Lower	3.44	9
Monocytes	0.54	0.78	0.14	0.23	Lower	1.47	7.5
Neutrophils	0.84	0.91	0.36	12.23	Upper	3.91	6.5
Platelets	0.58	0.87	0.22	129	Lower	2.20	5
Sex	0.53	0.56	0.07	0.5	Upper	0.38	10
Sodium	0.74	0.92	0.38	145.1	Upper	3.52	6
Total protein	0.98	0.89	0.35	61	Lower	5.73	7
Urea	0.95	0.92	0.44	10.9	Upper	5.43	8
WBC	0.86	0.88	0.32	13.5	Upper	3.87	7

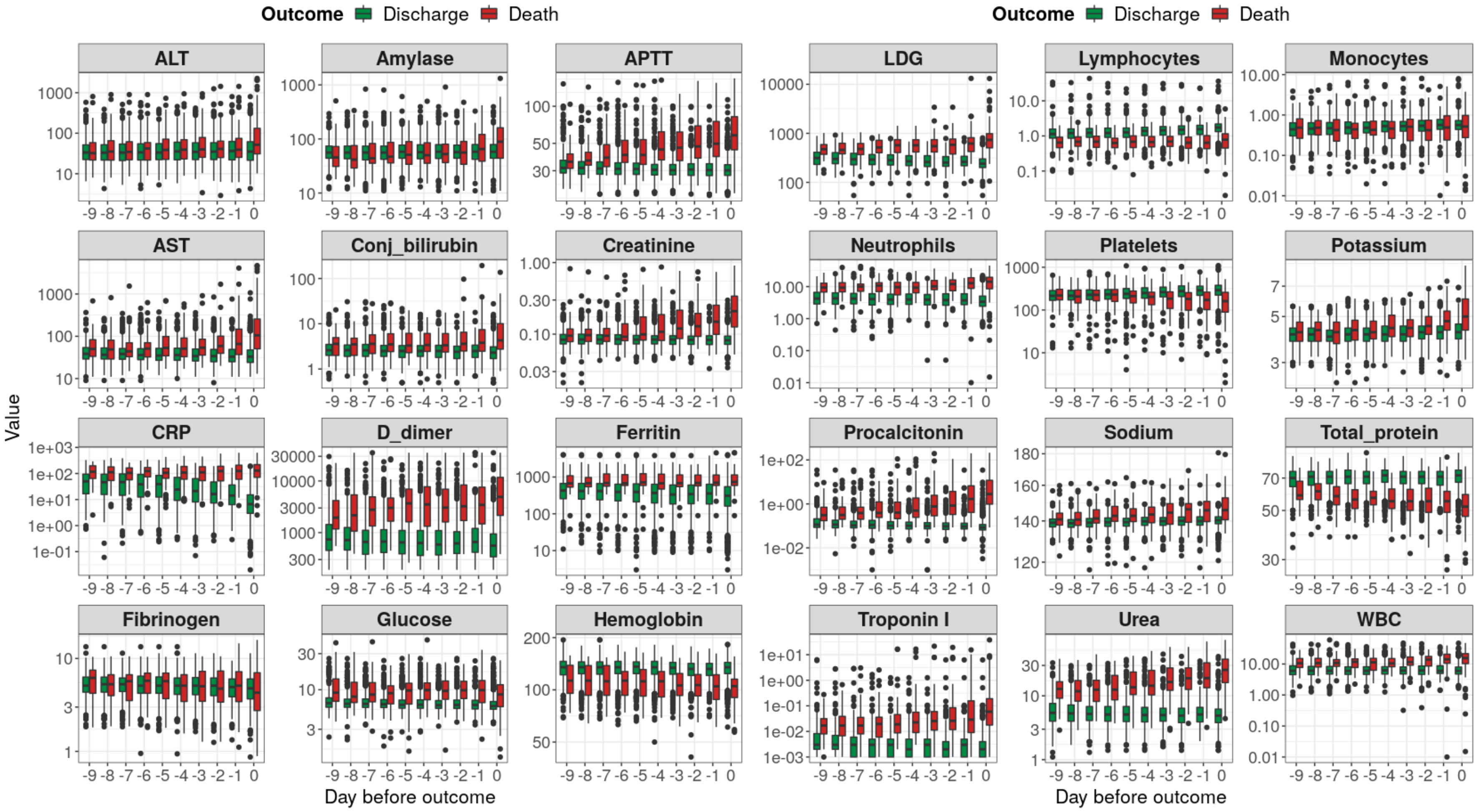

Supplementary Figure B. Time-varying features behavior before outcome (training cohort – wave 1)

C. Sensitivity analysis

A sensitivity analysis was performed in the following way. Using Wave II data, we followed the same steps as those described in Methods section: calculation of partial FIIs and prediction ranges for the available features, their filtering with the same bounds (FII ≥ 3, prediction range ≥ 7) and temporal validation. For validation, we used Wave I data. The partial characteristics of the features are given in Table C.1. The derived score is given in Table C.2, its validation results – in Fig. C.

Table C.1 Partial prediction effectiveness of the features (Wave II)

Feature	Sensitivity	Specificity	Precision	Threshold	Threshold Type	FII	Median prediction range, days
Age	0,61	0,78	0,17	69	Upper	1,69	13
Amylase	0,75	0,76	0,19	81	Upper	2,29	6
APTT	0,89	0,88	0,35	41,5	Upper	4,07	8
AST	0,77	0,83	0,24	70	Upper	2,76	6
BMI	0,22	0,84	0,08	23,45	Lower	0,37	13
Conj. bilirubin	0,86	0,76	0,21	3,4	Upper	2,97	8
Creatinine	0,78	0,90	0,35	0,12	Upper	3,42	5
CRP	0,85	0,79	0,23	117,67	Upper	3,03	9
D-dimer	0,87	0,90	0,38	2496	Upper	4,11	8
Glucose	0,89	0,85	0,31	10	Upper	3,83	7.5
Hemoglobin	0,75	0,90	0,35	109	Lower	3,32	7
Lymphocytes	0,74	0,91	0,38	0,5	Lower	3,36	9
Monocytes	0,52	0,91	0,30	0,15	Lower	2,39	7
Neutrophils	0,79	0,84	0,27	12,55	Upper	2,98	7
Platelets	0,61	0,93	0,38	120	Lower	2,99	7
Sex	0,51	0,53	0,07	0,5	Upper	0,17	12
Sodium	0,75	0,92	0,39	143,3	Upper	3,48	5
Total_protein	0,85	0,95	0,55	55	Lower	4,73	6
Urea	0,92	0,93	0,47	11,7	Upper	4,90	7
WBC	0,80	0,81	0,24	14,27	Upper	2,84	7

Table C.2 The proposed score components (Wave II)

Feature	Threshold	Threshold Type	FII
APTT	> 41,5	Upper	4
CRP	> 117,67	Upper	3
D-dimer	> 2496	Upper	4
Glucose	> 10	Upper	4
Hemoglobin	< 109	Lower	3
Lymphocytes	< 0,5	Lower	3
Urea	> 11,7	Upper	5
Total score:			26 max

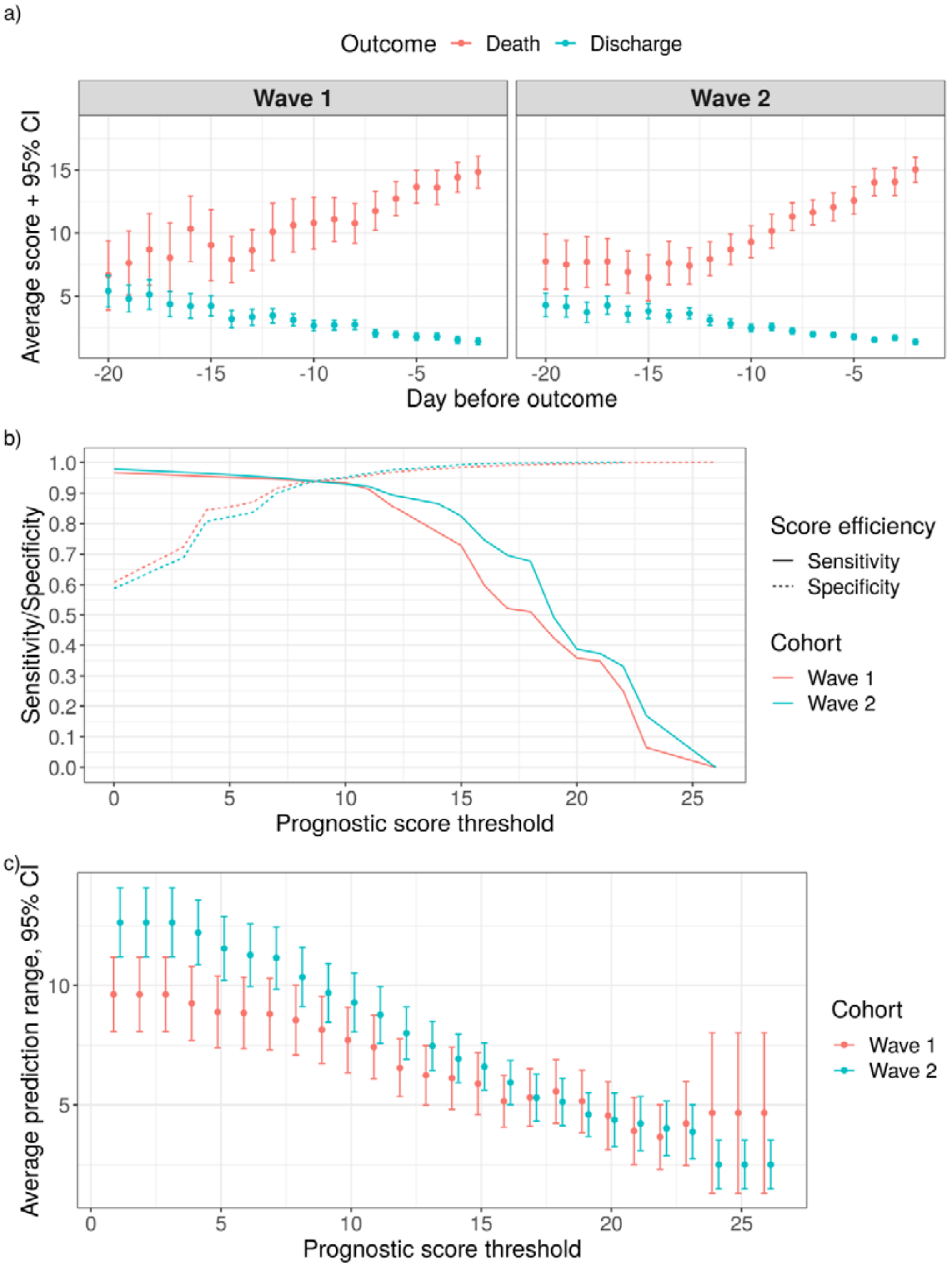

Supplementary Figure C. Results of score sensitivity analysis, points represent mean values, error bars – 95% confidence intervals for the mean values a) average score variation within three weeks before outcome: b) sensitivity/specificity trade-off for various threshold levels; c) prediction range dependency on a chosen threshold level

D. Subgroup analysis

In subgroup analysis we compared the score behavior for two age groups (≥65 y.o and <65 y.o). The resulting characteristics are represented in Figure D.

frontiers

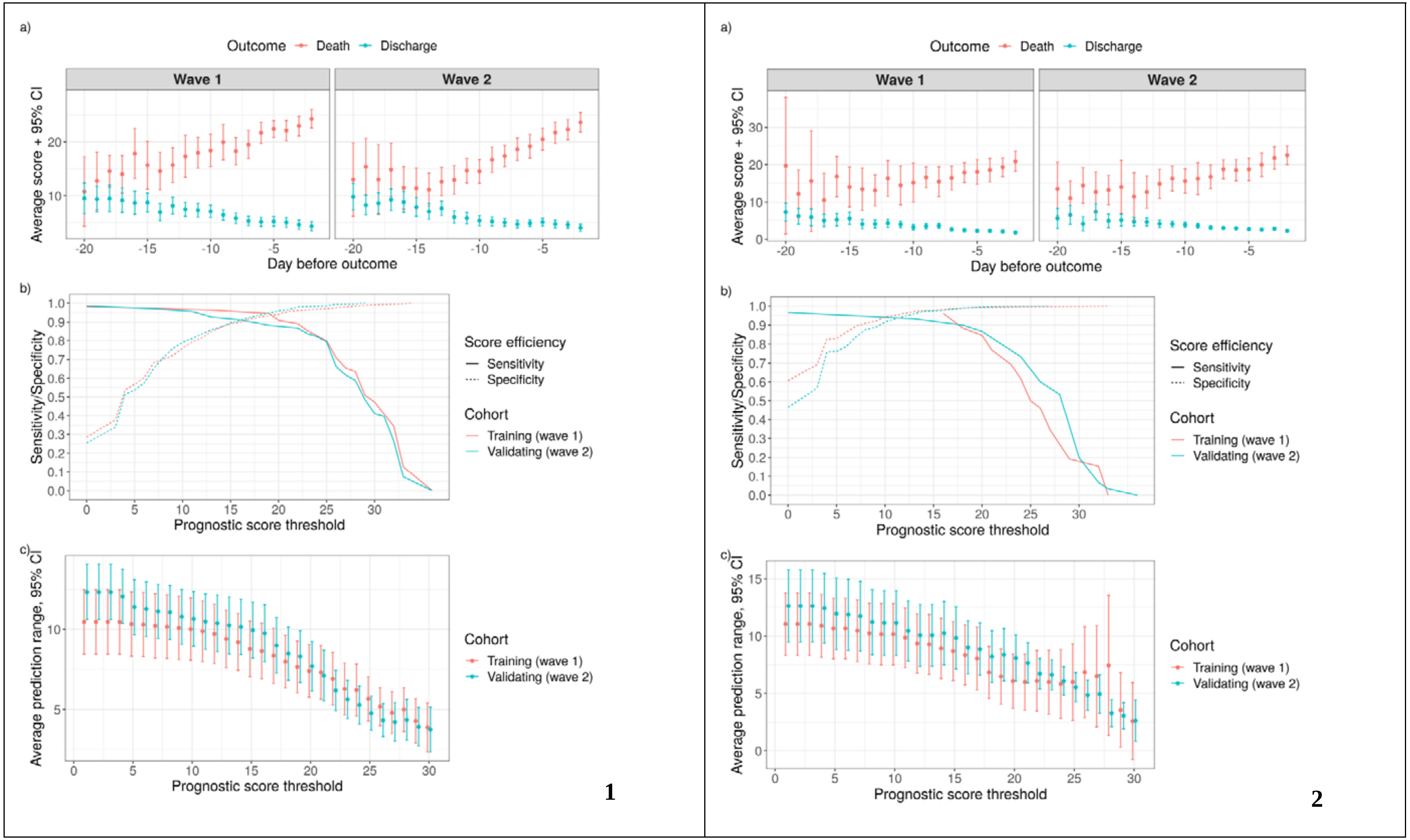

Supplement Figure D – The score characteristics for 1. older age group (≥65 y.o) and 2. younger age group (<65 y.o)

E. Comparison of conventional and experimental testing rates

Figure C represents a QQ plot with the score quartiles comparison between samples with an experimental (regular) and a conventional testing rate. A fitted linear regression line is depicted for the estimation of potential systematic biases introduced with a testing rate practice.

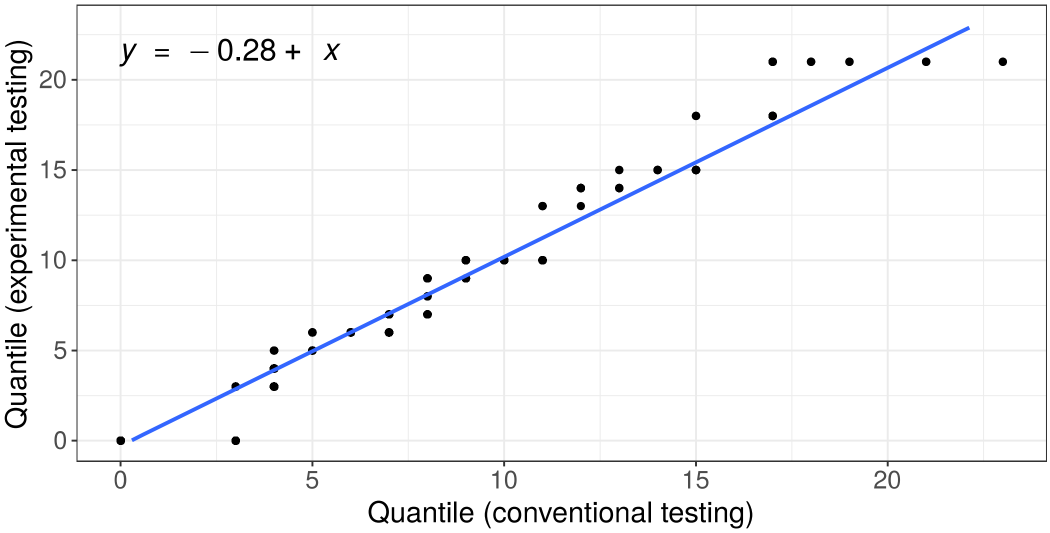

Supplementary Figure C. The comparison of score value distributions obtained by means of conventional (according to daily doctor decisions) and experimental (at least once per three days) testing strategies
